# Editorial: Liver diseases and programmed cell death

**DOI:** 10.3389/fphar.2023.1182013

**Published:** 2023-03-28

**Authors:** Peng Cao, Yongyi Liang, Yi Wang, Chao Mao, Weijia Wang, Sha Li, Lianxiang Luo

**Affiliations:** ^1^ Department of Pharmacy, Union Hospital, Tongji Medical College, Huazhong University of Science and Technology, Wuhan, China; ^2^ The First Clinical College, Guangdong Medical University, Zhanjiang, China; ^3^ Department of Critical Care Medicine, Sichuan Academy of Medical Science and Sichuan Provincial People’s Hospital, Chengdu, China; ^4^ Department of Experimental Radiation Oncology, The University of Texas MD Anderson Cancer Center, Houston, TX, United States; ^5^ State Key Laboratory of Cellular Stress Biology, School of Life Sciences, Xiamen University, Xiamen, Fujian, China; ^6^ School of Public Health, Shanghai Jiao Tong University, Shanghai, China; ^7^ The Marine Biomedical Research Institute, Guangdong Medical University, Zhanjiang, China

**Keywords:** non-alcoholic fatty liver disease, non-alcoholic steatohepatitis, alcohol-related liver disease, drug induced liver injury, hepatocellular carcinoma, programmed cell death

Given its indispensable status as an organ in the human body and its central role in human metabolism, liver disorders have become a subject of great interest among researchers worldwide. There has been a growing body of evidence indicating that the demise of hepatocytes plays a pivotal role in the development of liver disease. In particular, programmed cell death (PCD) in the epithelial cell subpopulation, may lead to disease progression as well as hepatocellular carcinogenesis (Luedde et al., 2014). As of now, programmed cell death encompasses a range of processes such as apoptosis, autophagy, ferroptosis, pyroptosis, necrosis, and more. In the past few years, there has been a surge in research on ferroptosis, a concept that was proposed 10 years ago and has gained significant attention from numerous scholars (Dixon et al., 2012). Ferroptosis has been defined as an iron-dependent form of cell death in which glutathione peroxidase 4 is a key regulatory protein (Stockwell, 2022). Several publications have reported that ferroptosis is associated with liver disease (Wu et al., 2021; Luo et al., 2021). The underlying mechanisms may be related to inflammatory responses, too (Deng et al., 2022). The present Research Topic is comprised of five primary articles and three evaluations that center on the Research Topic of PCD. The articles present innovative viewpoints on the interplay between liver disease and programmed cell death, which could potentially guide the advancement of liver disease therapies.

With regards to the subject matter, the three review articles center around non-alcoholic fatty liver disease (NAFLD), non-alcoholic steatohepatitis (NASH), and liver fibrosis. NAFLD is a chronic condition that can lead to the development of cirrhosis and hepatocellular carcinoma, and is often accompanied by complications such as cardiovascular disease (Brunt et al., 2015). The review by Tan et al. suggests that lipophagy plays an important role in maintaining the body’s metabolic homeostasis and that mTORC1 is a key regulator in this process. They also suggest that restoring lipid droplet autophagy in NAFLD by targeting mTORC1 may be an effective therapeutic strategy for NAFLD treatment. NASH, an advanced form of non-alcohol-associated fatty liver, is more likely to develop into cirrhosis and hepatocellular carcinoma (Xu et al., 2022). Xiong et al. reviewed NAFL from the perspective of ferroptosis mechanisms. They also discussed the relationship between mitochondrial dysfunction, ferroptosis, and NASH. In addition, potential targets for the treatment of non-alcoholic steatohepatitis were explored and proposed. Li and Zhu also reviewed the role of ferroptosis in the development of liver fibrosis, taking the mechanism of ferroptosis as a starting point. The difference is that they focused on the different types of drugs that induce ferroptosis in hepatic stellate cells. At the same time, they summarized the pharmacological effects of these drugs on hepatic fibrosis to provide new targets for the clinical treatment of hepatic fibrosis.

Within our Research Topic, there are 5 research papers that have been included. Chen et al. investigated the effect of intermittent hypoxia (IH) on the fatty liver by establishing a murine model. They found that IH exposure attenuated lipid accumulation, lipid peroxidation, neutrophil infiltration, and apoptotic processes induced by high fat and high sugar. More importantly, the pool of bile acids produced by IH was overall more likely to be FXR agonistic, which allowed IH to achieve therapeutic effects in the treatment of NAFLD. In contrast to Chen et al., Ye et al. analyzed the correlation between NAFLD and colorectal neoplasms. In the study, they found that NAFLD increased the risk of colorectal neoplasms. In addition, after bioinformatic analysis, they suggested that ferroptosis and cuproptosis may be involved in colorectal cancer associated with NAFLD. Cuproptosis is a copper-dependent death that occurs through the direct binding of copper to the lipidated components of the tricarboxylic acid cycle and was proposed in 2022 (Tsvetkov et al., 2022). For NAFLD, Gan Shuang granules may play a therapeutic role (Zhi et al.). It is derived from the classical Chinese formula Xiaoyao San, which has been reported to treat liver fibrosis, and depression (Jiao et al., 2021; Zhou et al., 2021). In recent years, severe liver disease can progress to hepatocellular carcinoma, a cancer with a 5-year survival rate of less than 20% (Moon et al., 2020). Zhou et al. constructed a prognostic model of coppering-related genes in hepatocellular carcinoma and performed immune infiltration, pantheon analysis, immune checkpoint analysis, and drug sensitivity analysis. The results showed that coppering-related genes, especially CDKN2A, could serve as potential prognostic predictors for HCC patients. Pyroptosis also plays an important role in hepatocellular carcinoma. Chen et al. established a prognostic signature for 24 pyroptosis-related genes, which provides a theoretical basis for risk stratification of hepatocellular carcinoma.

To summarize, the articles presented in this segment pertain to liver ailments that are associated with PCD-related liver diseases, and offer a promising approach for the management of liver disorders. Despite progress made in the treatment of fatty liver through intervention with insulin and hypoglycemic agents, there are still pressing issues that require immediate attention. One such issue is the absence of animal models to establish the efficacy of IH treatment at various time intervals, leaving the importance of duration unknown. Moreover, many studies have utilized data obtained from public databases that are inevitably skewed by the innate presence of diseases. Despite the significant effort required to translate the theoretical approach of manipulating cell death for the treatment of liver disease into clinical practice, its potential for success is highly encouraging.
